# Rheology-Guided and CFD-Integrated Analysis of Non-Isothermal Gelation Kinetics in a Three-Stage Cooling Die for Soy Protein Concentrate Extrusion

**DOI:** 10.3390/gels12040339

**Published:** 2026-04-17

**Authors:** Timilehin Martins Oyinloye, Won Byong Yoon

**Affiliations:** 1 Department of Food Science and Biotechnology, College of Agriculture and Life Sciences, Kangwon National University, Chuncheon 24341, Republic of Korea; oyinloyetm@kangwon.ac.kr; 2 Elder-Friendly Food Research Center, Agriculture and Life Science Research Institute, Kangwon National University, Chuncheon 24341, Republic of Korea; 3 Department of Food Biotechnology and Environmental Science, Kangwon National University, Chuncheon 24341, Republic of Korea

**Keywords:** viscoelasticity, structural development rate, Arrhenius analysis, thermal transition, protein aggregation, extrusion, non-isothermal gelation, cooling die

## Abstract

Soy protein concentrate (SPC) undergoes continuous thermal and structural changes during passage through a cooling die, yet these changes are often interpreted using viscosity-based descriptions that do not explicitly account for structural development rate (SDR). This study developed a rheology-guided framework to analyze SPC behavior in a three-stage cooling die by integrating isothermal and non-isothermal rheological characterization with computational fluid dynamics (CFD). SPC samples containing 76, 78, and 80% moisture were evaluated using strain sweep, frequency sweep, viscosity, time sweep, and temperature sweep tests. Lower moisture promoted stronger structure development, higher viscosity, and faster gelation. For the 76% moisture sample, peak SDR increased from 6.66 Pa/s at 50 °C to 22.46 Pa/s at 100 °C, while the time to peak decreased from 937 to 360 s. During non-isothermal cooling, the major structure development occurred in the 80–50 °C interval, where ΔG′ reached 4902.54 Pa at 76% moisture. CFD analysis showed that the gelation-kinetics-based model predicted both pressure and extrudate temperature more accurately than the viscosity-based model. Pressure RMSE ranged from 8.57 to 14.43 kPa for the kinetic model, compared with 11.31 to 22.39 kPa for the viscosity model. These results demonstrate that the three-stage cooling die should be interpreted as a coupled thermal, flow, and structure-development domain.

## 1. Introduction

Plant-based meat analogues have attracted substantial interest as the food industry seeks protein-rich products with a lower environmental burden while still meeting consumer expectations for meat-like texture, structure, and sensory quality [1,2,3]. Among the available structuring technologies, high-moisture extrusion (HME) is one of the most effective because it couples thermal denaturation, shear-induced alignment, phase deformation, and cooling-assisted structure fixation in a continuous process [3]. In such systems, final product quality is governed not only by formulation but also by how composition-dependent rheology interacts with process conditions such as moisture content, temperature history, and die design [1,4].

Soy proteins are widely used for high-moisture meat analogues (HMMA) because of their availability, balanced amino acid composition, and strong ability to form cohesive networks under thermomechanical treatment [5,6,7]. Soy protein concentrate (SPC), in particular, provides a protein-rich matrix suitable for structure development during extrusion, but its functional behavior remains highly dependent on thermal history, water availability, and the extent of intermolecular aggregation [6]. This becomes even more important in structured products that must retain dispersed oil or fat-like domains, because the protein matrix must remain flowable enough for transport and phase distribution while also strengthening sufficiently to immobilize the internal structure before extrusion [8].

In HME, the cooling die should not be viewed only as a thermal setting section. It is also the region where the protein melt continues to flow while undergoing progressive network formation, consolidation, and structure fixation under a changing temperature field [9]. Recent work on temperature-controlled cooling dies has shown that cooling conditions strongly influence anisotropic structure development and final textural quality [10,11]. In particular, temperature gradients along the cooling die alter rheological properties and the balance between matrix stiffness and molecular alignment, indicating that the die environment actively controls the transition from deformable melt to fixed structure [9]. In a three-stage cooling die, this transition is expected to be even more pronounced because each zone imposes a distinct thermal stage on a material whose properties are continuously evolving. Therefore, isothermal measurements alone are insufficient to describe SPC behavior in the cooling die. They capture structure development only at fixed temperatures. Non-isothermal characterization is needed to represent the continuous thermal transitions experienced during passage through the three-stage die.

As material properties change during extrusion, pressure behavior becomes a critical processing variable because it reflects the evolving resistance of the material under coupled thermal and flow conditions [12,13]. Die pressure is strongly influenced by moisture content, temperature history, and the structural state of the protein matrix, and extrusion studies have shown that protein-based systems respond sensitively to changes in torque, die pressure, and thermal conditions during processing [1,2,14]. In a staged cooling die, pressure measured near the inlet represents the initial resistance encountered as the hot SPC melt enters the cooling region, whereas pressure measured near the outlet reflects the cumulative effect of progressive cooling and structure development along the die. In this way, the inlet and outlet pressures provide complementary information on how the imposed thermal gradient influences local flow resistance, structural buildup, and discharge behavior. If gel-network development becomes excessive during passage through the die, pressure may increase substantially and impair stable extrusion; conversely, if structure development remains insufficient, the material may discharge before the internal structure is adequately stabilized. Therefore, inlet and outlet pressures are not merely mechanical readouts. They are practical indicators of how the staged cooling program governs processability, structure fixation, and extrusion stability.

Previous studies have already shown the value of rheology-informed and viscosity-based approaches for interpreting structure formation in HMMA, and viscosity-based predictive models have been used to relate protein behavior to extrusion outcomes [15]. Likewise, earlier SPC-related computational work has demonstrated that computational fluid dynamics (CFD) can be a useful framework for describing transport and structure-related phenomena in protein-based systems [16]. However, a viscosity-only interpretation becomes increasingly limited when the material is not merely cooling but simultaneously undergoing gelation, network strengthening, and structure fixation within the die. A viscosity description can represent resistance to flow at a given condition, but it does not directly resolve how rapidly the internal protein network develops or how that evolving structure modifies process behavior across successive cooling zones.

Rheological and kinetic analyses are therefore needed to provide a more mechanistic description of SPC behavior under cooling-die-relevant conditions [17,18]. Storage modulus (G′), loss modulus (G″), and their evolution during heating and cooling can track the transition from viscous-dominant melt to elastic network, while structure development rate (SDR), gelation temperature, and Arrhenius-type kinetic parameters provide direct information on the rate and thermal sensitivity of network formation [16,19,20]. Studies conducted under high-moisture-extrusion-relevant thermal histories have emphasized that rheological characterization under combined thermal and mechanical treatment is essential for clarifying the otherwise “black-box” behavior of protein extrusion [18]. For SPC in a three-stage cooling die, such a framework is especially important because moisture-dependent gelation kinetics are likely to determine when the material behaves predominantly as a flowing melt and when it begins to behave as a setting structure that resists further deformation.

Despite growing interest in cooling-die design, rheology-based prediction, and CFD analysis of plant-protein structuring, a clear gap remains in linking moisture-dependent SPC gelation kinetics with flow and pressure development inside a staged cooling die [9,15]. In particular, the transition from inlet flow to outlet discharge in a three-stage die has not been sufficiently interpreted by comparing a conventional viscosity-based description with a gelation-kinetics-based framework. Addressing this gap is important because it clarifies whether pressure evolution and discharge behavior are better explained by progressive network formation than by viscosity alone. To the best of our knowledge, this is one of the first studies to directly couple rheology-derived non-isothermal gelation kinetics, based on SDR and Arrhenius-type analysis, with CFD to interpret SPC behavior in a three-stage cooling die.

Therefore, this study aimed to establish a process-oriented framework for interpreting SPC behavior in a three-stage cooling die by integrating rheological characterization, gelation kinetics, and CFD-based analysis. The specific objectives were to: (i) characterize the moisture-dependent rheological behavior of SPC under isothermal and non-isothermal conditions using viscosity, G′, G″, and SDR; (ii) determine gelation-related kinetic parameters describing the onset, rate, and thermal sensitivity of structure formation; (iii) analyze flow and thermal behavior in the three-stage cooling die using CFD; and (iv) compare viscosity-based and gelation-kinetics-based interpretations of SPC behavior, with emphasis on inlet and outlet pressure as indicators of processability and structure fixation.

## 2. Results and Discussion

### 2.1. Rheological Characterization of SPC at Different Moisture Contents

**Figure 1:**
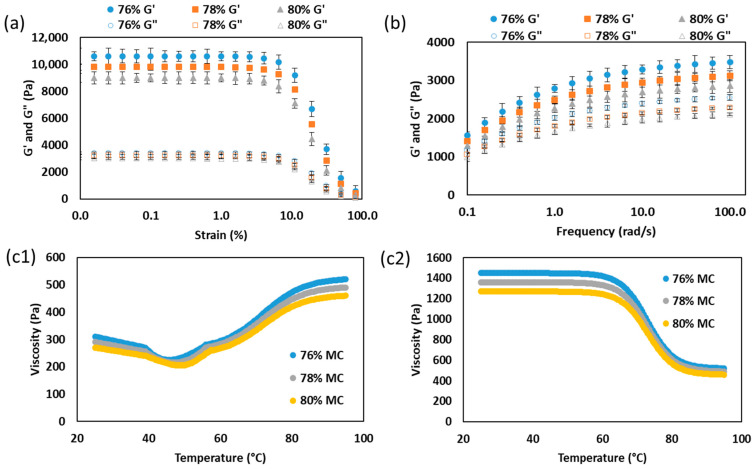
Rheological characterization of SPC samples prepared at 76, 78, and 80% moisture: (**a**) strain sweep profiles showing storage modulus (G′) and loss modulus (G″); (**b**) frequency sweep profiles; (**c1**) apparent viscosity during heating; and (**c2**) apparent viscosity during cooling.

The rheological behavior of SPC at different moisture contents is shown in Figure 1. In the strain sweep analysis (Figure 1a), all samples showed a distinct linear viscoelastic region (LVR) at low strain, where G′ remained higher than G″, indicating elastic-dominant weak-gel behavior before thermal treatment. Within this region, both moduli followed the order 76% > 78% > 80% moisture, showing that lower moisture promoted a stronger pre-gel structure. This trend is consistent with higher solids concentration at lower moisture, which increases protein–protein proximity and promotes intermolecular associations such as hydrophobic interactions, hydrogen bonding, and sulfhydryl–disulfide interchange. Similar concentration-dependent increases in soy-protein gel modulus were reported by Renkema and Van Vliet [21], who related higher protein content to denser and more load-bearing structures.

Beyond the linear region (>4.0%), both G′ and G″ decreased sharply with increasing strain, indicating disruption of the weak-gel network under large deformation. The higher-moisture samples softened earlier and more extensively, suggesting lower resistance to strain-induced breakdown. This behavior indicates that excess water acted as a plasticizing phase that increased intermolecular spacing and reduced junction density in the hydrated SPC matrix. A similar weakening effect of increasing water content has been reported for concentrated soy-protein systems under high-moisture processing conditions [13].

The frequency sweep results (Figure 1b) also confirmed gel-like behavior, as G′ remained higher than G″ over the entire angular-frequency range. Both moduli increased with frequency, indicating structured systems that still exhibited time-dependent relaxation. The consistently higher G′ and G″ values of the 76% moisture sample indicate a more coherent and less relaxation-sensitive network, whereas the 80% moisture sample remained the weakest.

The apparent viscosity profiles during heating and cooling (Figure 1(c1,c2)) provide additional information directly relevant to die flow. During heating, viscosity decreased with temperature for all moisture levels, consistent with thermal softening and increased molecular mobility. The lower-moisture sample maintained the highest viscosity throughout, confirming that water reduced flow resistance by diluting the protein network. During cooling, viscosity increased again as temperature decreased, reflecting structure rebuilding and gel-network development. This viscosity recovery was more pronounced at lower moisture, indicating that concentrated SPC systems regained flow resistance more rapidly. This transition reflects the behavior expected in the three-stage cooling die. SPC enters in a deformable state and progressively develops resistance to flow as gelation proceeds.

Overall, the strain, frequency, and viscosity results show that reduced moisture strengthened the SPC matrix and maintained higher flow resistance. These rheological differences provide the basis for the later comparison of the viscosity-based and gelation-kinetics-based models.

### 2.2. Isothermal Gelation Behavior of SPC at Different Temperatures and Moisture Contents

**Figure 2:**
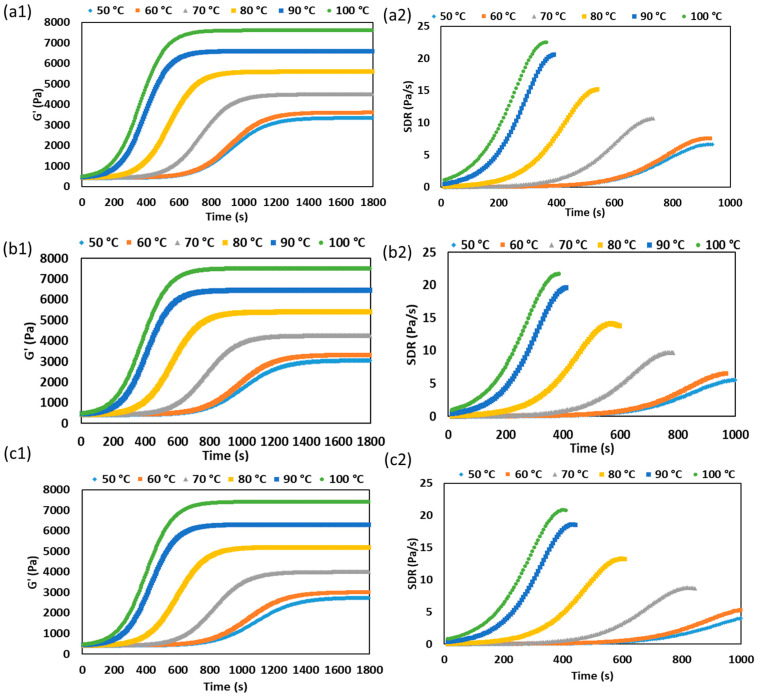
Time sweep profiles of storage modulus (G′) and corresponding structure development rate (SDR) of SPC at different temperatures for 76% (**a**), 78% (**b**), and 80% (**c**) moisture contents. Subfigures (**a1**,**b1**,**c1**) present the G′ profiles, whereas subfigures (**a2**,**b2**,**c2**) present the corresponding SDR profiles.

The isothermal gelation behavior of SPC at different temperatures is shown in Figure 2. For all moisture levels, G′ increased sigmoidally with time, while the SDR curves showed a single distinct peak, indicating rapid structure development followed by stabilization. As the temperature increased from 50 to 100 °C, the SDR peak increased and shifted to shorter times for all formulations, confirming faster gelation at higher temperatures. For the 76% moisture sample, peak SDR increased from 6.66 ± 0.51 Pa/s at 50 °C to 22.46 ± 0.62 Pa/s at 100 °C, while the time at peak decreased from approximately 937 s to 360 s. Similar trends were observed for the 78% and 80% moisture samples. At 78% moisture sample, peak SDR increased from 5.59 ± 0.48 to 21.64 ± 0.49 Pa/s and time to peak decreased from approximately 1018 s to 385 s, and at 80% moisture sample, peak SDR increased from 4.65 ± 0.39 to 20.85 ± 0.44 Pa/s and time to peak decreased from approximately 1092 s to 403 s. These results indicate that increasing temperature accelerated protein unfolding and subsequent network formation in SPC [22]. Similar behavior was reported by Li et al. [23], who found that preheating increased storage modulus and shortened gelation time in soy protein-stabilized gels.

Moisture content also affected isothermal structure development. At the same temperature, lower-moisture SPC consistently showed a higher SDR peak and a shorter time to peak than higher-moisture SPC. This suggests that reducing water content increased effective protein concentration and promoted earlier intermolecular junction formation through stronger hydrophobic interactions, hydrogen bonding, and sulfhydryl–disulfide interchange within the concentrated protein matrix [6,13]. Pietsch et al. [13] similarly reported that thermomechanical treatment altered SPC rheology through changes in protein proximity and interaction density.

A similar temperature-dependent acceleration of structure development has also been reported in xanthan gum-locust bean gum gels, where higher temperature shifted gelation to shorter times [20]. Although that system is polysaccharide-based, it supports the broader interpretation that a higher SDR peak reflects faster structure build-up under favorable thermal conditions. Overall, both increasing temperature and decreasing moisture promoted faster SPC gelation, with the 76% moisture sample showing the most rapid structure development.

### 2.3. Isothermal Gelation Kinetics of SPC at Different Moisture Contents

**Figure 3:**
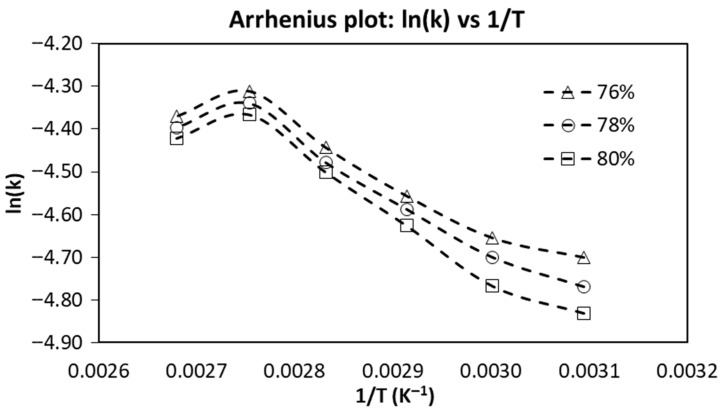
Arrhenius plots of the apparent isothermal gelation rate constant, ln(k), as a function of 1/T.

The Arrhenius plots of the apparent isothermal gelation rate constant are shown in Figure 3, and the corresponding kinetic parameters are summarized in Table 1. In all samples, ln(k) decreased as 1/T increased, indicating slower gelation at lower temperature. This is consistent with the time-sweep results, in which higher temperature produced larger SDR peaks and shorter times to peak, indicating faster network development. The slight upward deviation at the high-temperature end suggests that the Arrhenius relationship was not perfectly linear across the full range, likely because protein unfolding, aggregation, and network consolidation contributed differently as temperature increased [20,24]. Similar non-ideal Arrhenius behavior has been reported in thermally induced gel systems when more than one structural process contributes to modulus development over the fitted temperature range [20].

The apparent activation energy increased from 8.02 kJ/mol at 76% moisture to 8.79 kJ/mol at 78% and 9.73 kJ/mol at 80%, indicating that greater thermal input was required to drive gelation as moisture increased. This agrees with the rheological results, which showed that lower-moisture samples showed faster structure development and higher peak SDR values at the same temperature. Reducing moisture increased effective protein concentration and brought protein molecules into closer proximity, thereby facilitating the intermolecular interactions required for gel formation. Similar behavior has been reported for soy protein gels, where increasing protein concentration enhanced gel development and strengthened the resulting network by promoting protein–protein association during heating [21,24].

The pre-exponential factor, k_0_, also increased with moisture content, from 0.18 s^−1^ at 76% moisture to 0.22 s^−1^ at 78% moisture and 0.30 s^−1^ at 80% moisture. However, k_0_ should be interpreted together with Ea. In the present study, the higher k_0_ at higher moisture did not result in faster gelation because the corresponding increase in E_a_ imposed a stronger thermal barrier. Similar Arrhenius-based kinetic analyses have been used in food gel systems to compare the temperature sensitivity of structure development even when the gelation pathway is complex [25].

Although the Arrhenius model was useful here for comparative interpretation, SPC gelation is inherently a multi-step process involving protein unfolding, aggregation, and network formation. Therefore, the fitted kinetic parameters should be regarded as apparent values rather than intrinsic single-step constants. The isothermal E_a_ values obtained in this study (8.02–9.73 kJ/mol) are much lower than activation energies commonly reported for soy-protein denaturation or aggregation [21,24]. This difference arises because the present isothermal rate constant was not derived from the maximum slope of the sigmoidal G′(t) curve rather than from direct molecular denaturation or aggregation kinetics. Accordingly, the fitted E_a_ values should be interpreted as apparent rheological parameters that describe the temperature sensitivity of modulus development under the selected fitting framework.

Overall, the isothermal kinetic analysis shows that lower moisture favored faster SPC gelation and reduced the apparent activation energy of structure development, with the 76% moisture sample showing the most favorable kinetic behavior among the tested samples.

### 2.4. Non-Isothermal Rheological Behavior of SPC During Heating and Cooling

**Figure 4:**
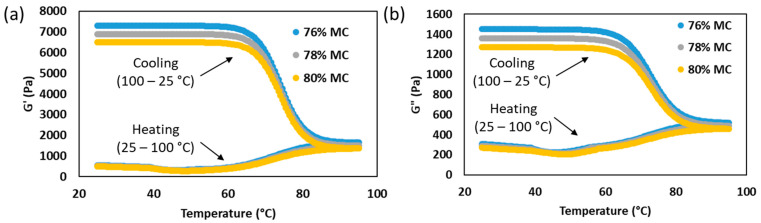
Temperature sweep profiles of (**a**) storage modulus (G′) and (**b**) loss modulus (G″) of SPC samples prepared at 76, 78, and 80% moisture during heating from 25 to 100 °C and subsequent cooling to 25 °C at 5 °C/min.

The non-isothermal rheological behavior of SPC at different moisture contents is shown in Figure 4. In all samples, both G′ and G″ decreased slightly during heating, with a local minimum appearing at approximately 45–48 °C, followed by a gradual increase as temperature approached the upper end of the sweep. This initial decrease is consistent with partial thermal softening of the hydrated protein matrix, where increased molecular mobility and weakening of pre-existing physical associations temporarily reduce viscoelastic resistance before extensive aggregation occurs [26].

The subsequent increase in modulus suggests that thermal unfolding progressively exposed reactive groups that later participated in network formation. In soy proteins, heating promotes exposure of hydrophobic regions and sulfhydryl groups, which enhances aggregation through hydrophobic interactions, hydrogen bonding, and disulfide bond formation. Renkema and Van Vliet [21] showed that heat denaturation is a prerequisite for soy protein gel formation, while O’Flynn et al. [26] reported that thermal treatment markedly altered the rheological behavior of soy protein dispersions. In the present study, the consistently higher G′ of the 76% moisture sample indicates that lower moisture increased effective protein concentration and favored earlier intermolecular junction formation [27].

A much stronger increase in G′ occurred during cooling than during heating, indicating that the major network fixation of SPC took place as the temperature decreased. This is important for the three-stage cooling die because SPC entered the die in a more deformable state and developed most of its elastic structure during progressive cooling, particularly between 70 and 80 °C. Therefore, the target extrudate temperature should remain within this range to prevent excessive pressure buildup in the cooling die. Similar cooling-dominant strengthening has been reported in soy protein systems; Isaschar-Ovdat et al. [28] described a two-stage modulus response in which final gel strengthening became much more evident during cooling.

Moisture content also affected the entire temperature-sweep response. The 76% moisture sample maintained the highest G′ and G″, followed by 78% and 80%, showing that reduced moisture produced a stronger and less relaxation-sensitive network. This agrees with recent plant-protein gel studies showing that higher solids content improves rheological strength by increasing interaction density and reducing dilution of the continuous matrix [24]. Overall, the temperature-sweep results show that SPC first underwent limited thermal softening during heating and then developed its main elastic network during cooling (70–80 °C), with lower moisture promoting stronger and earlier structure fixation.

### 2.5. Gelation Transition Temperature and Sectional Cooling Behavior

The heating-phase gelation transition parameters fitted over 34–51 °C and the sectional cooling results are summarized in Table 2 and Table 3, respectively. During heating, the fitted T_gel_ increased slightly from 47.02 to 48.31 °C with increasing moisture, while the corresponding G′ at T_gel_ decreased from 311.86 to 273.78 Pa. This indicates that higher moisture slightly delayed the onset of effective gel-network development and reduced structural strength at the transition point [13,21]. A similar composition-dependent shift in gelation temperature was reported by Yoon et al. [29], where moisture influenced the temperature at which the G′ minimum occurred and the material transitioned from thermal softening to gel formation.

The fitted polynomial coefficients also reflected the moisture effect. As moisture increased from 76 to 80%, the absolute values of β, α_1_, α_2_, and α_3_ all decreased, indicating that the heating-phase modulus transition became less temperature-sensitive at higher moisture. This behavior is consistent with dilution of the protein matrix, which increases intermolecular spacing and weakens temperature-driven protein–protein association [13,26]. Renkema and Van Vliet [21] similarly reported that soy-protein gelation depends strongly on concentration and interaction balance, with more concentrated systems showing clearer thermal transitions. In the present study, the lower coefficient magnitudes at higher moisture are therefore consistent with the higher T_gel_ and lower G′ values at transition.

The cooling-stage analysis showed that most structure development occurred in Stage II (80–50 °C) for all moisture levels. At 76% moisture, ∆G′ in Stage II reached 4902.54 Pa, compared with 742.98 Pa in Stage I and only 2.67 Pa in Stage III. The same pattern was observed at 78 and 80% moisture, confirming that the main gel network developed in the intermediate cooling region rather than immediately after die entry or near die exit. A similar interval-dominant non-isothermal gelation response was reported for xanthan gum–locust bean gum gels [20].

Moisture also influenced the intensity of structure development during cooling. In Stage II, ∆G′ decreased from 4902.54 Pa at 76% moisture to 4451.34 Pa at 80%, while the maximum dG′/dt decreased from 33.22 to 30.00 Pa/s, respectively (Table 3). This shows that lower moisture not only promoted earlier gelation during heating but also enhanced the rate and extent of network build-up during cooling. Similar behavior has been reported for SPC during high-moisture processing, where stronger protein–protein interactions improved structure fixation under thermomechanical treatment [13].

Overall, increasing moisture slightly shifted T_gel_ to higher temperature and reduced the modulus at transition, whereas the major cooling-induced structure development consistently occurred in Stage II. These results identify the central cooling section as the dominant zone of gel-network formation and show that lower-moisture SPC undergoes earlier and stronger structure fixation. This stage-dependent behavior provides a basis for interpreting how viscosity-based and gelation-kinetics-based CFD models predict flow resistance and pressure development in the three-stage cooling die.

### 2.6. Non-Isothermal Gelation Kinetics During the Principal Cooling Stage

**Figure 5:**
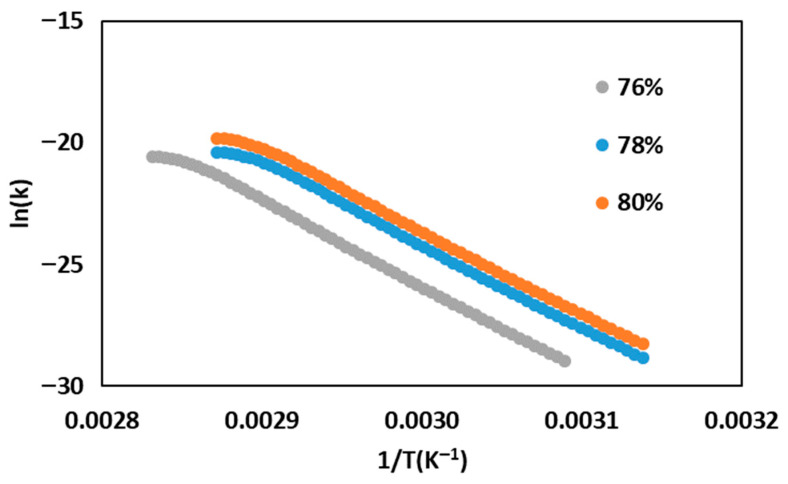
Arrhenius plots of the apparent non-isothermal gelation rate constant, ln(k), as a function of 1/T for SPC samples prepared at 76, 78, and 80% moisture.

The non-isothermal gelation kinetics of SPC during the principal cooling stage are presented in Figure 5 and Table 4. The Arrhenius plots showed good linearity for all moisture levels (R^2^ ≥ 0.99), indicating that structure development in the selected cooling region was well described by the linearized Arrhenius relationship. This confirms that the main increase in G′ during Stage II can be treated as the dominant gelation window for kinetic comparison among the three formulations. Similar rheology-based non-isothermal analyses have been used to quantify gelation during thermal transitions in surimi and hydrocolloid systems, where the principal region of modulus increase was taken as the relevant kinetic domain.

The apparent activation energy (E_a_) decreased slightly with increasing moisture, from 287.00 to 279.82 kJ/mol. This suggests that, within the principal cooling-stage interval, the apparent thermal barrier for cooling-induced structure development became marginally lower at higher moisture. This ordering differs from the isothermal results, which is not surprising because the present values were obtained under progressive cooling rather than fixed-temperature holding. In non-isothermal systems, the fitted E_a_ reflects the combined effects of temperature change, molecular mobility, and structural reorganization within a defined interval rather than a single intrinsic gelation event. Yoon et al. [29] similarly reported moisture-dependent changes in non-isothermal E_a_ for protein systems during thermal aggregation, while Zhang et al. [20] showed that cooling-stage gelation kinetics in xanthan gum-locust bean gum mixtures were strongly region-dependent.

The pre-exponential factor (k_0_) did not show a simple monotonic trend with moisture, varying from 5.40 × 10^33^ at 76% moisture to 2.40 × 10^33^ at 78% moisture and 3.77 × 10^33^ at 80% moisture. Such variation is common in Arrhenius analysis because k_0_ is derived from the regression intercept and is closely linked to the fitted Ea. Therefore, k_0_ should be interpreted together with E_a_, rather than independently. The lower ln(k) values of the 76% moisture sample across the cooling-stage plot do not indicate weaker gel formation overall, but rather a different kinetic response within the selected cooling interval.

Overall, SPC gelation during the main cooling stage followed an apparent Arrhenius-type relationship within the selected fitting interval, while moisture caused only modest differences in the apparent thermal barrier governing structure fixation. However, this interpretation should be treated with caution because protein gelation is not a single-step event, but a coupled process involving unfolding, aggregation, and network consolidation. Therefore, the present Arrhenius treatment is used as a practical comparative framework rather than a complete mechanistic description. For future work, alternative approaches such as model-free isoconversional analysis or multi-step kinetic fitting may provide a more rigorous description of the evolving rate-limiting steps under non-isothermal conditions.

### 2.7. Pressure Prediction and Experimental Validation in the Three-Stage Cooling Die

**Figure 6:**
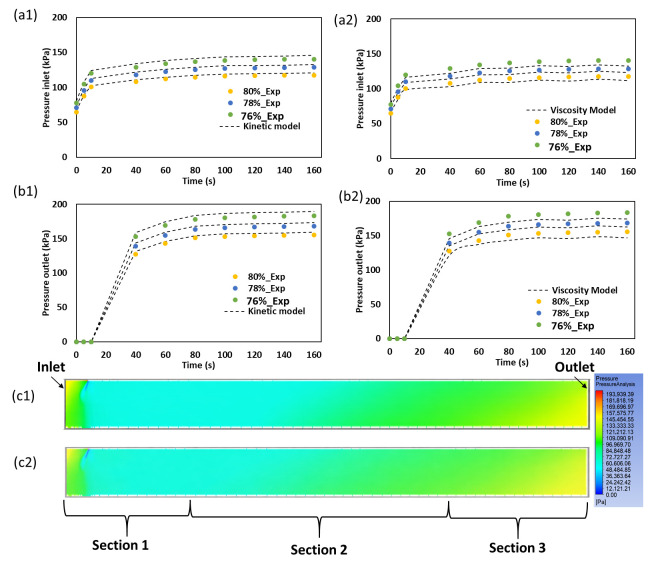
Pressure prediction and experimental validation in the three-stage cooling die for SPC samples prepared at 76, 78, and 80% moisture. Predicted and experimental pressure at the die inlet for the gelation-kinetics-based model (**a1**) and viscosity-based model (**a2**), and at the die outlet for the gelation-kinetics-based model (**b1**) and viscosity-based model (**b2**). Pressure contour distributions along the cooling die for the 80% moisture sample comparing the gelation-kinetics-based model (**c1**) and viscosity-based model (**c2**). Flow direction (inlet to outlet) and cooling Sections 1, 2, and 3 are indicated in the contour plots.

The pressure prediction results are shown in Figure 6. In both models, pressure increased with extrusion time at both measurement positions, and the outlet pressure remained higher than the inlet pressure. This indicates that flow resistance developed progressively along the cooling die as the SPC melt cooled and became more structured. Experimentally, the highest pressure was observed at 76% moisture, followed by 78% and 80% moisture, at both the inlet and outlet. This trend agrees with the rheological results and indicates greater flow resistance at lower moisture.

A clear difference was observed between inlet and outlet behavior. At the inlet, pressure increased rapidly during the early stage and then approached a near-steady level. At the outlet, the increase was delayed but much larger in magnitude. This shows that the major pressure build-up occurred after the melt had progressed through the cooling die. The melt entered the die at high temperature and with relatively low resistance. It then cooled progressively and developed a more rigid structure toward the exit. Previous studies on cooling dies in high-moisture extrusion have likewise shown that die temperature profile and geometry strongly influence pressure development and final structuring [1,14].

The gelation-kinetics-based model showed consistently lower RMSE values than the viscosity-based model, indicating closer agreement with the experimental data. For the kinetic model, RMSE values were 14.43 and 13.99 kPa at 76% moisture, 10.91 and 10.72 kPa at 78% moisture, and 8.58 and 8.57 kPa at 80% moisture for the inlet and outlet, respectively. The corresponding values for the viscosity model were higher, namely 20.59 and 22.39 kPa at 76% moisture, 11.31 and 13.40 kPa at 78% moisture, and 13.34 and 19.95 kPa at 80% moisture. These results indicate that the kinetic model captured the temporal pressure evolution more effectively, particularly near the outlet where structure fixation became more important. The pressure contours support this interpretation by showing a gradual pressure rise along the die length and a stronger increase toward the outlet region.

The better comparative performance of the kinetic model is consistent with the rheological results of this study. Earlier sections showed that major structure development occurred during cooling, especially in the central cooling region. A viscosity-only model can represent temperature-dependent flow resistance, but it cannot explicitly account for progressive gel-network formation. By contrast, the kinetic model incorporates evolving structure development and is therefore better suited to the continuously changing conditions inside the cooling die. Similar coupling between cooling, rheology, and pressure development has been emphasized in extrusion studies of plant proteins [1,16].

Overall, the pressure results show that the three-stage cooling die should be interpreted as a coupled flow and structure domain. The improved agreement of the kinetic model with the experimental data demonstrates that incorporating gelation development provides a more suitable basis for analyzing pressure evolution, processability, and structure fixation during SPC extrusion.

### 2.8. Temperature Prediction and Experimental Validation of SPC Extrudate

**Figure 7:**
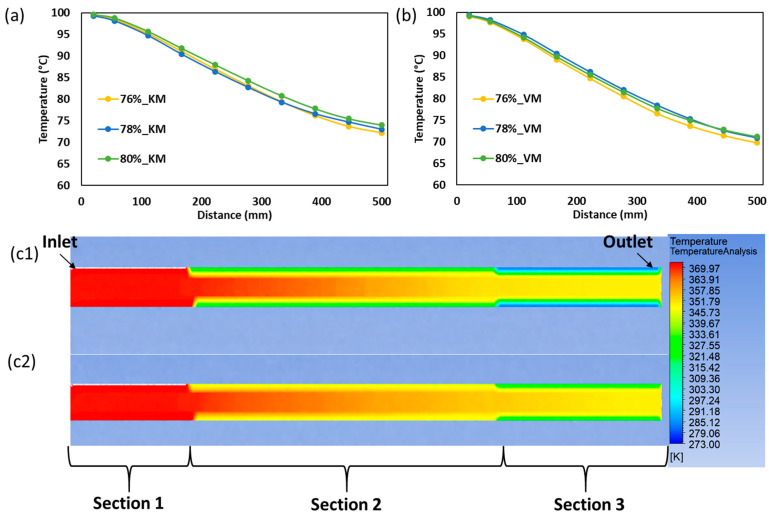
Temperature prediction and experimental validation of SPC extrudate in the cooling die. Axial temperature profiles predicted by the gelation-kinetics-based model (**a**) and viscosity-based model (**b**) for SPC samples prepared at 76, 78, and 80% moisture. Temperature contour plots for the 80% moisture sample comparing the gelation-kinetics-based model (**c1**) and viscosity-based model (**c2**). Flow direction (inlet to outlet) and cooling Sections 1, 2, and 3 are indicated in the contour plots.

The temperature prediction results are shown in Figure 7. In both models, melt temperature decreased progressively along the cooling die, from about 100 °C at the inlet to about 72–74 °C at the outlet, consistent with the imposed three-stage cooling profile and the expected function of the die in reducing melt temperature while allowing structure fixation. Similar cooling-die behavior has been reported in high-moisture extrusion studies, where gradual thermal reduction generates viscosity gradients that contribute to structure development [1].

A key advantage of the CFD analysis is that it provides the full axial temperature profile inside the cooling die, whereas the experimental measurement was practically limited to the extrudate temperature at the die exit. For this reason, model accuracy was evaluated using the outlet temperature only, not the entire simulated temperature field. This distinction is important because both models were compared against the same experimentally accessible discharge condition, while the internal temperature gradients remained available only through simulation. The CFD results therefore provide additional process information that cannot be obtained directly from the experiment, particularly the location and magnitude of the thermal drop along the die.

Although both models reproduced the overall cooling trend, the kinetic model showed closer agreement with the experimental exit temperature. The RMSE values for the kinetic model were 0.36, 0.21, and 0.06 °C for 76%, 78%, and 80% moisture, respectively, compared with 0.43, 0.51, and 1.12 °C for the viscosity model. This indicates that the viscosity-based approach was adequate for describing the general cooling pattern, but the kinetic model more closely captured the coupled thermal and structural evolution of SPC during passage through the die. This agrees with the rheological results of the present study, which showed that major structure development occurred during cooling rather than heating. Similar coupling between thermal history and structure development has been reported in soy-protein systems, where final gel strengthening becomes more pronounced during cooling [21,28].

The simulated temperature profiles also showed that the main temperature drop occurred through the die walls towards the central region of the die especially in cooling Section 2. This is important because that region was identified as the main structure-development zone from the rheological and sectional cooling analyses. Comparable extrusion studies have also linked axial temperature gradients in cooling dies to viscosity change and anisotropic structure formation [1,13].

Overall, the temperature results confirm that CFD was useful not only for validating the exit condition, but also for revealing the internal thermal profile that governs structure fixation inside the die. The lower outlet-temperature RMSE of the kinetic model shows that it is more suitable for describing the continuously changing SPC state during staged cooling, supporting the overall objective of comparing viscosity-based and gelation-kinetics-based models for interpreting flow behavior and process development in the three-zone cooling die.

## 3. Conclusions

This study established a rheology-guided framework for interpreting SPC behavior in a three-stage cooling die by integrating isothermal and non-isothermal rheological analysis with CFD simulation. Lower moisture consistently strengthened the SPC network, as shown by the higher G′, G″, and viscosity of the 76% moisture sample compared with 78% and 80% moisture, indicating greater resistance to deformation and higher flow resistance before and during cooling. Under isothermal conditions, increasing temperature accelerated gelation, with the peak SDR of the 76% moisture sample increasing from 6.66 Pa/s at 50 °C to 22.46 Pa/s at 100 °C, while the time to peak decreased from about 937 to 360 s. The isothermal Arrhenius analysis further showed that the apparent activation energy increased from 8.02 to 9.73 kJ/mol as moisture increased, confirming that lower-moisture SPC gelled more readily. Under non-isothermal conditions, the fitted gelation transition temperature increased slightly from 47.02 to 48.31 °C with increasing moisture, while the main structure development occurred during Stage II (80–50 °C), where ∆G′ reached 4902.54, 4685.62, and 4451.34 Pa for 76%, 78%, and 80% moisture, respectively. The cooling-stage kinetic analysis also showed Arrhenius-type behavior, with apparent E_a_ values of 287.00, 280.14, and 279.82 kJ/mol for 76%, 78%, and 80% moisture, respectively. CFD simulation showed that both pressure and temperature evolved progressively along the cooling die, but the gelation-kinetics-based model provided better agreement with experiment than the viscosity-based model. For pressure prediction, the kinetic-model RMSE ranged from 8.57 to 14.43 kPa, compared with 11.31 to 22.39 kPa for the viscosity model, while for extrudate temperature, the kinetic-model RMSE was only 0.36, 0.21, and 0.06 °C for 76%, 78%, and 80% moisture, respectively. From a practical perspective, the results suggest that careful control of Stage 2 (80–50 °C) is especially important for stable pressure development and effective product structuring in industrial cooling-die design. Overall, the three-stage cooling die should be interpreted as a coupled thermal, flow, and structure-development domain.

## 4. Materials and Methods

### 4.1. Sample Preparation

Soy protein concentrate (SPC), containing approximately 69% protein (dry basis) and <8% moisture (Shandong Wonderful Biotech Co., Ltd., Dongying, China), was used to evaluate moisture-dependent gelation. SPC samples were prepared at 76%, 78%, and 80% moisture (wet basis) by mixing the powder with distilled water to a constant total mass. Mixtures were homogenized for 2 min using a high-shear Stephan cutter mixer (UMC-5 Electronic, Stephan Machinery GmbH, Hameln, Germany) until a homogeneous paste was obtained. To ensure uniformity, entrapped air was removed under vacuum, and samples were equilibrated at room temperature before rheological testing, ensuring that the observed differences in gelation behavior were primarily attributable to moisture content.

### 4.2. Extrusion and Cooling-Die System

**Figure 8:**
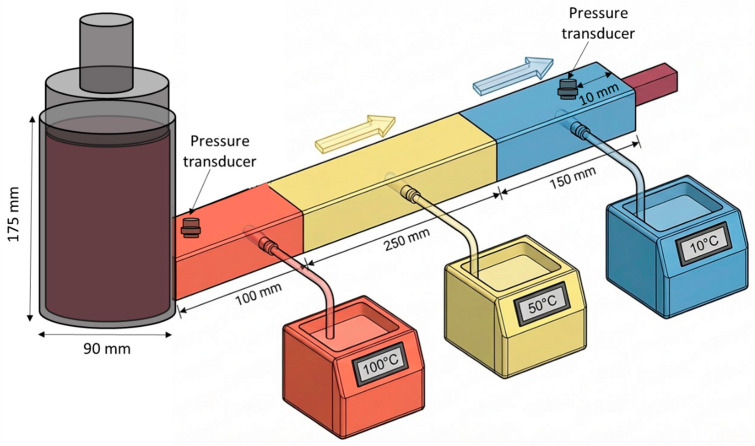
Schematic representation of the extrusion setup.

Extrusion was carried out using a laboratory-scale piston-driven assembly designed to allow independent control of melt compression, die temperature profile, and pressure monitoring (Figure 8). The SPC paste was loaded into a cylindrical stainless-steel barrel with an inner diameter of 90 mm and a height of 175 mm, and then compressed using a texture analyzer (TA.XT Plus, Stable Micro Systems, Surrey, UK) fitted with a matching piston. The piston displacement rate was fixed at 1 mm/s, corresponding to a volumetric flow rate of 6.51 × 10^−7^ m^3^/s, to maintain a steady flow of SPC through the barrel and cooling die.

The barrel was thermally conditioned at 100 °C using an external water-bath system to ensure that the SPC entered the die in a molten and deformable state. The melt then passed into a rectangular cooling die with a cross-section of 25 mm × 10 mm. The die was divided into three independently controlled sections arranged in series, with lengths of 100, 250, and 150 mm for Sections 1, 2, and 3, respectively (Figure 8). The temperatures of the three sections were set at 100, 50, and 10 °C, respectively, to impose a controlled axial temperature gradient along the die.

Pressure development during flow through the cooling die was monitored using two compact pressure transducers connected to an Arduino-based data acquisition system (Model M3200, TE Connectivity, Schaffhausen, Switzerland). One transducer was installed 10 mm downstream from the cooling-die inlet to monitor pressure near the die entrance, while the second was positioned 10 mm upstream of the die outlet to measure the pressure before extrusion from the die.

The three-stage cooling design was selected to provide progressive structure development rather than abrupt cooling in a single step. Section 1 was intended to preserve melt flowability immediately after barrel discharge. Section 2 provided intermediate cooling to promote gradual network formation, while Section 3 supplied stronger cooling to stabilize the structure before discharge. Under these conditions, the temperature of the extruded melt at the die outlet was maintained between 70 and 75 °C to ensure consistent gelation behavior at the exit.

The present piston-driven system was selected to isolate cooling-die flow, thermal evolution, and structure fixation under controlled inlet conditions. However, it does not fully reproduce the complex thermo-mechanical history of industrial twin-screw extrusion, where screw configuration, shear, mixing intensity, residence-time distribution, and specific mechanical energy all influence melt development before entry into the cooling die. Therefore, the present results should be interpreted primarily as a controlled analysis of post-barrel cooling-die behavior rather than a full representation of industrial HME.

### 4.3. Rheological Measurements

Rheological measurements were performed using a Discovery Hybrid Rheometer HR-3 (TA Instruments, New Castle, DE, USA) equipped with a Peltier temperature control system. A parallel-plate geometry (40 mm in diameter) and a fixed gap of 1.0 mm were used. To minimize moisture loss during measurement, a solvent trap was applied to the Peltier plate. Samples were loaded carefully to avoid excessive pre-shearing and allowed to equilibrate for 3 min before measurement.

Oscillatory strain sweep tests were conducted to determine the linear viscoelastic region (LVR). The strain amplitude was increased logarithmically from 0.01 to 100% at a constant angular frequency of 1 rad/s. The LVR was defined as the strain range over which the storage modulus (G′) and loss modulus (G″) remained within ±5% of their peak values and the oscillatory conditions used in the subsequent rheological tests were selected within this range to ensure that the measured response reflected the intrinsic structure of the sample without causing structural breakdown [16]. Frequency sweep tests were conducted within the LVR over the angular frequency range of 0.1–100 rad/s to confirm the viscoelastic characteristics of the hydrated SPC systems and to verify the stability of the network response under small-amplitude oscillatory shear. Viscosity was determined in a separate measurement during heating from 25 to 100 °C and subsequent cooling back to 25 °C at a constant rate of 5 °C/min, using a fixed shear condition (0.05%), to evaluate the temperature-dependent flow behavior of SPC as a function of moisture content. All rheological measurements were performed using independently prepared sample batches, and each condition was tested in three or more replicates; the reported trends were interpreted based on the consistency of these repeated measurements.

### 4.4. Isothermal Gelation Analysis

The isothermal rheological behavior of SPC was evaluated using time sweep measurements at fixed temperatures (50, 60, 70, 80, 90, and 100 °C). For each test, the sample was heated from 25 °C to the target temperature at 10 °C/min and then held isothermally for 30 min, while the viscoelastic response was recorded as a function of time. These tests were used to compare the extent and rate of gel network development under constant thermal conditions. The structure development rate (SDR) was calculated from the first derivative of G′ with respect to time [20]:

(1) $$SDR=\frac{{dG}^{\prime }}{dt},$$

where SDR is the structure development rate (Pa/s), G′ is the storage modulus, and t is time (s). The maximum value of the SDR curve was taken as the peak structure development rate, and the corresponding time was defined as the time required to reach the maximum rate of gelation.

To compare isothermal gelation kinetics among temperatures, an apparent gelation rate constant (k) was estimated from the maximum slope of the sigmoidal G′(t) curve. Assuming logistic-type modulus development, the maximum slope at the inflection point is related to the total modulus increase and the apparent rate constant as follows [30,31]:

(2) $$k=\frac{{4SDR}_{peak}}{G_{\infty }^{\prime }-G_{0}^{\prime }},$$

where SDRpeak is the peak structure development rate (Pa/s), G′_0_ is the initial storage modulus (Pa), and G′_∞_ is the final plateau storage modulus (Pa). The term (G′_∞_ − G′_0_) represents the total modulus increase during isothermal gelation. In this study, k was treated as an apparent rheological rate constant derived from the sigmoidal development of G′(t). Therefore, it represents the temperature sensitivity of modulus buildup under the selected fitting framework rather than an intrinsic molecular denaturation or aggregation constant.

The values of k obtained at different temperatures were transformed to ln(k) and plotted against 1/T for Arrhenius analysis, from which the apparent activation energy (E_a_) and pre-exponential factor (k_0_) were determined. Because SPC gelation involves coupled structural events such as unfolding, aggregation, and network formation, the kinetic parameters obtained from this analysis were interpreted as apparent values for comparative evaluation of temperature sensitivity rather than as single-step mechanistic constants.

### 4.5. Non-Isothermal Temperature Sweep Analysis

Non-isothermal rheological analysis was performed to replicate the continuously changing thermal conditions experienced by SPC in the cooling die during high-moisture extrusion. Temperature sweep tests were conducted by heating the samples from 25 to 100 °C and subsequently cooling them back to 25 °C at a constant rate of 5 °C/min [32]. Throughout the thermal cycle, the viscoelastic response was monitored continuously under small-amplitude oscillatory shear within the LVR, using a strain of 0.05% and an angular frequency of 1 rad/s. The heating and cooling stages were evaluated separately to distinguish thermal softening and transition behavior during heating from gel network development during cooling.

### 4.6. Determination of Gelation Transition and Sectional Cooling Behavior

To relate the rheological response of SPC to the thermal history imposed by the three-stage cooling die, the temperature sweep data were analyzed separately for the heating and cooling phases. During heating, the gelation temperature (T_gel_) was defined as the temperature corresponding to the minimum in the fitted G′–temperature curve, which marks the transition from thermally softened behavior to the onset of gel network development. To determine this point, the temperature dependence of G′ around the heating transition region was fitted using a cubic polynomial model following the thermorheological approach described by Yoon et al. [29]:

(3) $$G^{\prime }=\beta +\alpha_{1}T+\alpha_{2}T^{2}+\alpha_{3}T^{3},$$

where G′ is the storage modulus (Pa), T is temperature (°C), β is the intercept term, and α_1_,α_2_, and α_3_ are the linear, quadratic, and cubic coefficients, respectively. In this model, β represents the baseline term of the fitted response, α_1_ describes the first-order temperature effect, α_2_ accounts for curvature in the relationship, and α_3_ represents higher-order nonlinearity in the thermal response.

The gelation temperature (T_gel_) was obtained from the first derivative of the polynomial with respect to temperature:

(4) $$\frac{dG^{\prime }}{dT}=\alpha_{1}+2\alpha_{2}T+{3\alpha }_{3}T^{2},$$

The value of T at which $\frac{dG^{\prime }}{dT}=0$ was taken as the stationary point of the fitted curve. The physically meaningful root within the fitted temperature range was selected as T_gel_. The second derivative of the polynomial was also evaluated to confirm that the selected point corresponded to a minimum in the fitted curve.

Because the SPC melt entered the cooling die at approximately 100 °C and then cooled progressively through three independently controlled sections, the cooling phase was analyzed in relation to the three die stages rather than by polynomial fitting. The cooling curve was divided into three temperature regions corresponding to the thermal progression of SPC in the die: Stage I (100–80 °C), Stage II (80–50 °C), and Stage III (<50 °C). For each stage, representative rheological parameters were extracted from the G′–temperature response. Stage I was used to describe the onset of structure development immediately after the melt entered the die, Stage II represented the main region of gel network development, and Stage III described the final stabilization of the structure before die exit. This stage-based analysis was adopted because the cooling curve primarily reflected progressive modulus build-up rather than a modulus minimum, and was therefore better described by sectional rheological parameters than by polynomial coefficients. The heating-phase parameters were used to evaluate the effect of moisture content on gelation transition, while the cooling-stage parameters were used to assess sequential structure development and stabilization under staged cooling conditions.

### 4.7. Non-Isothermal Gelation Kinetics During Cooling

The non-isothermal gelation kinetics of SPC were evaluated from the cooling phase of the temperature sweep, since gel network development and structure fixation occurred progressively as the material passed through the three-stage cooling die. The kinetic analysis was therefore performed over the temperature interval in which G′ increased during cooling, corresponding to the principal region of structure development. The rate of structure development was treated as a first-order process with respect to the storage modulus [20,33]:

(5) $$\frac{dG^{\prime }}{dt}={kG}^{\prime },$$

where k is the apparent first-order rate constant (s^−1^) and t is time (s). The temperature dependence of k was described using the Arrhenius equation:

(6) $$k=k_{0}\;exp\left(-\frac{E_{a}}{RT}\right),$$

where k_0_ is the pre-exponential factor (s^−1^), E_a_ is the apparent activation energy (J mol^−1^), R is the universal gas constant (8.314 J·mol^−1^·K^−1^), and T is the absolute temperature (K). By combining Equations (5) and (6), the following linearized form was obtained:

(7) $$ln\left(\frac{1}{G^{\prime }}\frac{{dG}^{\prime }}{dt}\right)=lnk_{0}-\frac{E_{a}}{R}\left(\frac{1}{T}\right),$$

where $ln\left(\frac{1}{G^{\prime }}\frac{{dG}^{\prime }}{dt}\right)$ is the logarithmic kinetic term used for linear regression against 1/T. The apparent activation energy was determined from the slope of the fitted line:

(8) $$slope=-\frac{E_{a}}{R},$$

and thus,

(9) $$E_{a}=-(slope)\times R,$$

The intercept of the regression line corresponds to $\ln k_{0}$, from which the pre-exponential factor can be obtained as:

(10) $$k_{0}=exp(intercept),$$

For each moisture level, the kinetic analysis was used to estimate the apparent activation energy and pre-exponential factor describing non-isothermal gelation during the principal cooling stage. The fitted values were E_a_ = 287.00, 280.14, and 279.82 kJ mol^−1^, and k_0_ = 5.40 × 10^33^, 2.40 × 10^33^, and 3.77 × 10^33^ s^−1^ for the 76%, 78%, and 80% moisture samples, respectively. Because SPC gelation is a complex multi-step transformation, the kinetic parameters obtained in the present study were interpreted as apparent values for comparative analysis. More advanced model-free isoconversional methods or multi-step model-fitting approaches may be applied in future studies to better resolve the contribution of overlapping structural events during non-isothermal gelation.

### 4.8. Computational Fluid Dynamics Modeling of SPC Flow in the Three-Stage Cooling Die

Three-dimensional CFD simulations were performed using ANSYS Fluent 2024 R2 (ANSYS Inc., Canonsburg, PA, USA) to analyze the flow and thermal behavior of SPC in the three-stage cooling die. Two constitutive approaches were compared. Model A used the experimentally measured temperature-dependent viscosity of SPC, whereas Model B incorporated non-isothermal gelation kinetics to account for progressive structure development during cooling. The objective was to compare the ability of both approaches to predict pressure development and temperature evolution in the cooling die.

#### 4.8.1. Governing Equations

The flow was treated as incompressible and non-isothermal. Conservation of mass, momentum, and energy was expressed as:

(11) ∇·u=0,

(12) $$\rho \left(\frac{\partial u}{\partial t}+u\cdot \nabla u\right)=-\nabla p+\nabla \cdot \tau ,$$

(13) $$\rho C_{p}\left(\frac{\partial T}{\partial t}+u\cdot \nabla T\right)=\nabla \cdot \left(k_{t}\nabla T\right)+\Phi ,$$

where u is the velocity vector (m s^−1^), ρ is the density (kg m^−3^), p is the pressure (Pa), T is temperature (K), C_p_ is the specific heat (J kg^−1^ K^−1^), k is thermal conductivity (W m^−1^ K^−1^), Φ is the viscous dissipation term (W m^−3^), and τ is the extra stress tensor (Pa) [34]. The stress tensor was written as:

(14) $$\tau =2\mu_{eff}D,$$

where μ_eff_ is the local effective viscosity (Pa·s) used in the CFD momentum equation, and D is the rate-of-deformation tensor:

(15) $$D=\frac{1}{2}\left(\nabla u+(\nabla {u)}^{T}\right),$$

The corresponding scalar shear rate was defined as:

(16) $$\dot{\gamma }=\sqrt{2D:D},$$

where $\dot{\gamma }$ is the shear rate (s^−1^) [35].

To account for the thermal response of SPC, the experimentally fitted temperature-dependent specific heat capacity and thermal conductivity were incorporated into the energy equation. For the 76%, 78%, and 80% moisture samples, the specific heat capacity was expressed as 0.0329T^2^ − 2.4683T + 3660.6, 0.0281T^2^ − 2.3489T + 3647.5, and 0.0346T^2^ − 3.0849T + 3644.3, respectively, where T is the temperature °C and C_p_ is in J kg^−1^ K. Similarly, thermal conductivity was expressed as −1 × 10^−5^T^2^ + 0.0005T + 0.5142, −5 × 10^−6^T^2^ − 0.0002T + 0.5081, and −2 × 10^−6^T^2^ − 0.0003T + 0.4900, respectively, where T is the temperature °C and kt is in W m^−1^ K.

#### 4.8.2. Geometry, Mesh, and Boundary Conditions

**Figure 9:**
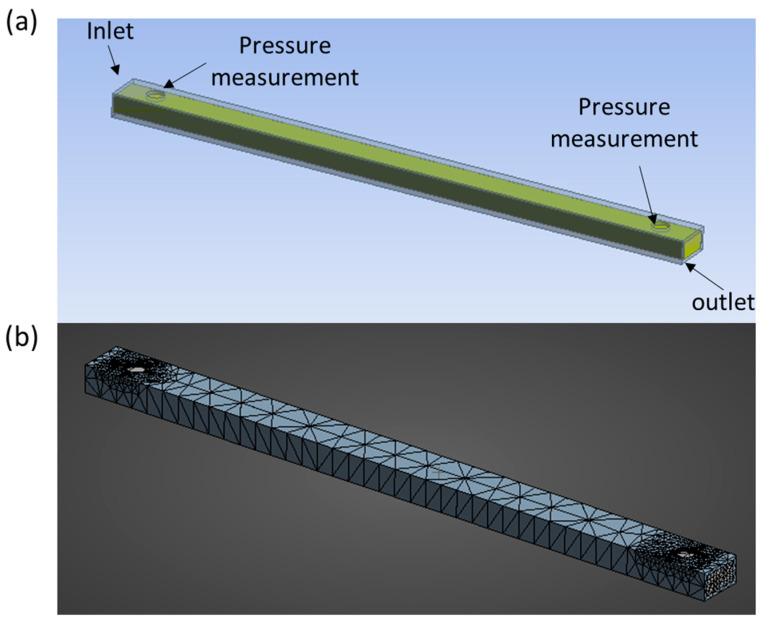
Computational model of the three-stage cooling die used for CFD analysis: (**a**) simplified cooling-die geometry showing the inlet, outlet, and pressure-transducer locations; and (**b**) structured tetrahedral mesh.

To reduce computational cost, the CFD domain was limited to the three-stage cooling die because the SPC temperature in the cylindrical barrel was maintained at 100 °C before entering the die. Therefore, only the rectangular cooling die was modeled for flow and heat-transfer analysis (Figure 9). The mesh was generated in ANSYS Meshing using an unstructured tetrahedral grid with local refinement near the die inlet, pressure-monitoring locations, and die outlet. A mesh-independence study was performed using three grid levels: coarse (115,650 nodes, 132,125 elements), medium (202,387 nodes, 258,460 elements), and fine (289,124 nodes, 375,152 elements). The predicted inlet pressure, outlet pressure, and outlet temperature changed noticeably from the coarse to the medium mesh, but the differences between the medium and fine meshes were less than 2.1%, 1.95%, and 1.3%, respectively. Therefore, the medium mesh was selected as a suitable compromise between numerical accuracy and computational cost. The die-wall temperatures were set at 100, 50, and 10 °C for Sections 1, 2, and 3, respectively. The inlet was defined as a velocity inlet based on the piston-driven volumetric flow rate, and the outlet was defined as a pressure outlet at 0 Pa gauge. All walls were treated as no-slip boundaries. Pressure values were extracted at two axial positions corresponding to the experimental transducer locations: 10 mm downstream of the die inlet and 10 mm upstream of the die outlet.

#### 4.8.3. Viscosity-Based Model

In Model A, the local effective viscosity was taken directly from the experimentally measured viscosity during cooling from 100 to 25 °C at 5 °C min^−1^. Because the material entered the die at elevated temperature and underwent progressive cooling, the viscosity used in the CFD model was implemented as a temperature-dependent function:

(17) $$\mu_{eff}=\mu_{\upsilon }(T),$$

where μ_eff_ is the apparent viscosity measured experimentally as a function of temperature (Pa s), and T is the local material temperature (K). In this model, viscosity varied only with temperature and did not include an explicit contribution from gelation progress.

#### 4.8.4. Gelation-Kinetics-Based Model

In Model B, the local structural state of SPC was represented by a dimensionless gelation variable, α, defined as:

(18) $$\alpha =\frac{G^{\prime }-G_{0}^{\prime }}{G_{\infty }^{\prime }-G_{0}^{\prime }},$$

where α is the gelation index (dimensionless), G′ is the local storage modulus (Pa), $G_{0}^{\prime }$ is the initial storage modulus at the start of cooling (Pa), and $G_{\infty }^{\prime }$ is the final storage modulus associated with the stabilized structure (Pa). Thus, α = 0 corresponds to the ungelled state and α = 1 corresponds to the fully developed gel structure. In Equation (18), $G_{0}^{\prime }$ and $G_{\infty }^{\prime }$ were determined from the non-isothermal cooling curve for each moisture level. Specifically, $G_{0}^{\prime }$ was taken as the storage modulus at the beginning of the selected cooling interval, while $G_{\infty }^{\prime }$ was taken as the storage modulus at the end of the cooling interval where the structure approached stabilization. This definition was adopted so that the gelation index represented the progressive structural development occurring under cooling-die-relevant non-isothermal conditions.

Using the first-order kinetic model from Section 4.7, the local modulus evolution during cooling was expressed as:

(19) $$\frac{{dG}^{\prime }}{dt}=k_{0}\exp\left(-\frac{E_{a}}{RT}\right)G^{\prime },$$

Substituting Equation (18) into Equation (19) gives the Eulerian transport form for the gelation variable:

(20) $$\frac{\partial \alpha }{\partial t}+u\cdot \nabla \alpha =\frac{1}{G_{\infty }^{\prime }-G_{0}^{\prime }}\frac{{dG}^{\prime }}{dt},$$

which becomes

(21) $$\frac{\partial \alpha }{\partial t}+u\cdot \nabla \alpha =k_{0}exp\left(-\frac{E_{a}}{RT}\right)\left[\alpha +\frac{G_{0}^{\prime }}{G_{\infty }^{\prime }-G_{0}^{\prime }}\right],$$

where the source term has a unit of s^−1^ [36,37]. The source term for the gelation variable, α, was implemented in ANSYS Fluent using a User-Defined Function (UDF). In this approach, the UDF was used to define the source term of the transported scalar based on the experimentally derived gelation behavior, allowing the local structural state to evolve during the CFD calculation as a function of the imposed thermal history. The resulting α field was then used to update the local effective viscosity in the gelation-kinetics-based model. This implementation was selected because it provides a flexible and standard route in Fluent for coupling experimentally derived constitutive or kinetic relations with the transport equations solved in the CFD domain.

The local effective viscosity in the kinetic model was linked to the evolving rheological state through the complex viscosity derived from oscillatory data:

(22) $$\left|\eta^{*}\right|=\frac{\sqrt{{G^{\prime }}^{2}+{G^{\prime \prime }}^{2}}}{\omega },$$

where $\left|\eta^{*}\right|$ is the complex viscosity (Pa·s), G′ is the storage modulus (Pa), G″ is the loss modulus (Pa), and ω is the angular frequency (rad s^−1^). In the present work, ω = 1 rad s^−1^, consistent with the temperature-sweep measurement. To relate oscillatory rheology to effective flow resistance, the Cox–Merz approximation was adopted [38,39]:

(23) $$\mu_{eff}\approx \left|\eta^{*}\right|\;\mathrm{at}\;\dot{\gamma }=\omega ,$$

where μ_eff_ is the local effective viscosity (Pa·s) and $\dot{\gamma }$ is the shear rate (s^−1^). The Cox–Merz relationship is widely used as an empirical approximation to relate complex viscosity from oscillatory measurements to apparent viscosity from steady shear, although exact agreement is material-dependent [38,39]. In this form, the viscosity used by the CFD solver increased not only because of cooling, but also because of progressive gel-network development predicted from the kinetic model.

#### 4.8.5. Numerical Procedure and Model Evaluation

Pressure-velocity coupling was handled with the SIMPLE algorithm, and second-order spatial discretization was applied for momentum and energy. Convergence was monitored through normalized residuals, with target values of 10^−5^ for continuity and momentum and 10^−6^ for energy. In addition to residual convergence, the predicted inlet pressure, outlet pressure, and outlet temperature were monitored to confirm solution stability. The simulations were performed under transient conditions over a physical simulation time of 200 s using an adaptive time-step scheme with a minimum time step of 1.0 × 10^−5^ s, a maximum time step of 1.0 s, and a maximum of 10 iterations per time step, until the monitored output variables no longer changed appreciably.

For model evaluation, the simulated pressure at the two transducer positions and the predicted outlet temperature were compared with the corresponding experimental measurements. Model performance was quantified using the root mean square error (RMSE):

(24) $$RMSE=\sqrt{\frac{1}{N}\sum_{i=1}^{N}{\left(y_{sim,i}-y_{exp,i}\right)}^{2}},$$

where y_sim,i_ is the simulated value, y_exp,i_ is the experimental value, and N is the number of comparison points. Separate RMSE values were calculated for pressure (Pa) and temperature (°C). RMSE was used in this study as a descriptive metric to compare predictive agreement between models and experimental observations. Because the model outputs were deterministic and evaluated against limited experimental datasets at fixed conditions, the RMSE differences were interpreted comparatively and were not subjected to formal statistical significance testing.

## Figures and Tables

**Table 1:** Arrhenius kinetic parameters for isothermal SPC gelation at different moisture contents.

	Moisture Content (%)		
	76%	78%	80%
Slope	−964.75	−1057.25	−1170.75
Intercept	−1.73	−1.50	−1.21
R^2^	0.9162	0.9302	0.9298
E_a_ (kJ/mol)	8.02	8.79	9.73
k_0_ (s^−1^)	0.18	0.22	0.30

**Table 2:** Heating-phase gelation transition parameters of SPC at different moisture contents.

Moisture	β	α_1_	α_2_	α_3_	R^2^	T_gel_ (°C)
76%	−10,580.83	857.72	−21.70	0.18	0.97	47.02
78%	−9509.27	765.10	−19.16	0.16	0.98	47.68
80%	−8229.31	660.05	−16.40	0.13	0.99	48.31

**Table 3:** Sectional cooling behavior of SPC at different moisture contents.

Moisture (%)	Stage	Start G′ (Pa)	End G′ (Pa)	∆G′ (Pa)	Peak dG′/dt (Pa/s)
	I	886.10 ± 18.42 ^a^	1629.08 ± 24.17 ^a^	742.98 ± 16.85 ^a^	3.05 ± 0.08 ^a^
76	II	1769.65 ± 27.33 ^a^	6672.19 ± 61.24 ^a^	4902.54 ± 54.18 ^a^	33.22 ± 0.74 ^a^
	III	7297.33 ± 68.21 ^a^	7301.02 ± 66.95 ^a^	2.67 ± 0.09 ^a^	0.22 ± 0.01 ^a^
	I	841.62 ± 17.56 ^b^	1551.73 ± 22.84 ^b^	710.11 ± 15.92 ^b^	2.88 ± 0.07 ^b^
78	II	1684.55 ± 25.41 ^b^	6370.17 ± 58.63 ^b^	4685.62 ± 49.77 ^b^	31.64 ± 0.69 ^b^
	III	6897.42 ± 64.35 ^b^	6904.05 ± 63.88 ^b^	2.58 ± 0.08 ^a^	0.21 ± 0.01 ^a^
	I	793.94 ± 16.91 ^c^	1468.54 ± 21.73 ^c^	674.60 ± 14.63 ^c^	2.73 ± 0.06 ^c^
80	II	1581.46 ± 23.68 ^c^	6032.80 ± 55.92 ^c^	4451.34 ± 46.28 ^c^	30.00 ± 0.63 ^c^
	III	6497.43 ± 60.14 ^c^	6503.27 ± 59.67 ^c^	2.57 ± 0.08 ^a^	0.20 ± 0.01 ^a^

Values are presented as mean ± standard deviation (n = 3). Different lowercase letters within the same column and the same cooling stage indicate significant differences among moisture contents at *p* < 0.05.

**Table 4:** Arrhenius kinetic parameters for non-isothermal SPC gelation during the principal cooling stage at different moisture contents.

	Moisture Content (%)		
	76%	78%	80%
Slope	−34,520.19	−33,694.38	−33,656.75
Intercept	77.67	76.86	77.31
R^2^	0.994	0.998	0.998
E_a_ (kJ/mol)	287.00	280.14	279.82
k_0_ (s^−1^)	5.40 × 10^33^	2.40 × 10^33^	3.77 × 10^33^

## Data Availability

The data presented in this study are available in the article. Further inquiries can be directed to the corresponding author.

## Abbreviations

The following abbreviations are used in this manuscript:

CFD	Computational fluid dynamics
HME	High-moisture extrusion
HMMA	High-moisture meat analogues
RMSE	Root mean square error
SDR	Structural development rate
SPC	Soy protein concentrate
LVR	Linear viscoelastic region
